# Embryonic rat vascular smooth muscle cells revisited - a model for neonatal, neointimal SMC or differentiated vascular stem cells?

**DOI:** 10.1186/2045-824X-6-6

**Published:** 2014-03-15

**Authors:** Eimear Kennedy, Roya Hakimjavadi, Chris Greene, Ciaran J Mooney, Emma Fitzpatrick, Laura E Collins, Christine E Loscher, Shaunta Guha, David Morrow, Eileen M Redmond, Paul A Cahill

**Affiliations:** 1 Vascular Biology and Therapeutics Laboratory, School of Biotechnology Faculty of Science and Health, Dublin City University, Dublin 9, Ireland; 2 Immunomodulation Research Group, School of Biotechnology Faculty of Science and Health, Dublin City University, Dublin 9, Ireland; 3 Department of Surgery, University of Rochester Medical Center, Rochester, NY 14642, USA

## Abstract

**Background:**

The A10 and A7r5 cell lines derived from the thoracic aorta of embryonic rat are widely used as models of non-differentiated, neonatal and neointimal vascular smooth muscle cells in culture. The recent discovery of resident multipotent vascular stem cells within the vessel wall has necessitated the identity and origin of these vascular cells be revisited. In this context, we examined A10 and A7r5 cell lines to establish the similarities and differences between these cell lines and multipotent vascular stem cells isolated from adult rat aortas by determining their differentiation state, stem cell marker expression and their multipotency potential *in vitro.*

**Methods:**

Vascular smooth muscle cell differentiation markers (alpha-actin, myosin heavy chain, calponin) and stem cell marker expression (Sox10, Sox17 and S100β) were assessed using immunocytochemistry, confocal microscopy, FACS analysis and real-time quantitative PCR.

**Results:**

Both A10 and A7r5 expressed vascular smooth muscle differentiation, markers, smooth muscle alpha - actin, smooth muscle myosin heavy chain and calponin. In parallel analysis, multipotent vascular stem cells isolated from rat aortic explants were immunocytochemically myosin heavy chain negative but positive for the neural stem cell markers Sox10^+^, a neural crest marker, Sox17^+^ the endoderm marker, and the glia marker, S100β^+^. This multipotent vascular stem cell marker profile was detected in both embryonic vascular cell lines in addition to the adventitial progenitor stem cell marker, stem cell antigen-1, Sca1^+^. Serum deprivation resulted in a significant increase in stem cell and smooth muscle cell differentiation marker expression, when compared to serum treated cells. Both cell types exhibited weak multipotency following adipocyte inductive stimulation. Moreover, Notch signaling blockade following γ-secretase inhibition with DAPT enhanced the expression of both vascular smooth muscle and stem cell markers.

**Conclusions:**

We conclude that A10 and A7r5 cells share similar neural stem cell markers to both multipotent vascular stem cells and adventitial progenitors that are indicative of neointimal stem-derived smooth muscle cells. This may have important implications for their use in examining vascular contractile and proliferative phenotypes *in vitro.*

## Introduction

The arterial blood vessel is comprised of three distinct layers; an innermost monolayer of endothelial cells, a medial layer composed primarily of vascular smooth muscle cells [vSMCs] [1-3] and some multipotent vascular stem cells [MVSCs] [1,4,5], and an outer adventitial layer of fibroblast cells and some vSMC-related stem cell antigen-1 (Sca1^+^) positive progenitor cells [6,7]. The medial vSMCs in the vessel wall are not terminally differentiated but can undergo “phenotypic switching” following vascular injury [8,9]. In a similar manner, vSMCs in culture are thought to be ‘phenotypically modulated’ [10,11,3]. These ‘contractile’ cells initially express markers for SMC differentiation such as smooth muscle α-actin (SMA), smooth muscle myosin heavy chain (SM-MHC), calponin (CNN1) and SM-22α. However, proliferating vSMC display the characteristics of de-differentiated vSMCs in that they have minimal expression of SMC differentiation marker proteins and do not contract when cultured on a silicone substrate [12]. Moreover, proliferating ‘synthetic’ vSMC can be converted back or ‘modulated’ to a more mature phenotype by placing the cells in quiescent media to selectively increase in the expression of the SMC differentiation markers [12,13].

Original studies in canine carotid arteries suggested that ‘neointimal modulated proliferative SMC’ were not derived from differentiated SMC but instead formed from a myosin negative type II medial SMC cell [14]. Recent lineage tracing studies *in vivo* using SM-MHC as a marker suggest that SM-MHC^-^ negative resident multipotent vascular stem cells [MVSCs], and not de-differentiated vSMCs, repopulate the neointima following vascular injury and proliferate and differentiate into vSMCs [5,15]. Moreover, Notch activation following co-culture of MVSCs with OP9-Delta1 feeder cells for 2 weeks promoted MVSC transition to vSMC [5]. MVSCs are resident stem cells located in the tunica media and adventitial layers of the arterial wall and express the neural crest cell marker Sox10, endoderm marker Sox17, glial cell marker S100β and neural filament-medium polypeptide (NFM) [5]. Sox10 is routinely used to identify and trace MVSCs in blood vessels [5,15]. MVSCs can be cloned from single cells, possess telomerase activity and can differentiate into Schwann cells, peripheral neurons, vSMCs, chondrocytes, adipocytes and osteoblasts [5].

The A10 and A7r5 cell lines were originally derived from the thoracic aorta of 14-17 day old embryonic BD1X rats and are a commonly used model of vSMC in culture [16]. Initial characterisation of these cells suggested that they were non-differentiated vSMC that differ from neonatal but bear significant resemblance to neointimal cells [16]. The functionality of A10 and A7r5 cells and their relevance to mechanisms underlying the contractile properties of highly differentiated vascular smooth muscle cells is questionable. Nevertheless, these cell lines exhibit an adult smooth muscle phenotype and show expression and promoter activity of several highly restricted smooth muscle cell markers [17]. Moreover, a phenotypic transition from vascular smooth to skeletal muscle and a detailed examination of the gene expression program associated with this transition has been reported [18]. The cells also have the ability to contract by both calcium- dependent and -independent mechanisms [19]. On the other hand, the actin cytomatrix of these cells shows many structural similarities to fibroblasts, much like other smooth muscle cell types that revert to a less differentiated phenotype in culture [1,16,17]. Despite this, the cell lines are widely used by researchers due to their apparent similarities to neointimal cells and therefore offer an excellent model system for studying the transcriptional regulation of vSMC markers and signaling cascades involved in neointima formation [16,17].

In light of the recent characterization of resident vascular stem cells within vascular medial and adventitial regions and their transition to vSMC following vascular injury [5,20], it has been suggested that traditionally defined proliferative/synthetic vSMCs, such as A10 and A7r5 cell lines may be derived from the differentiation of resident stem cells in culture rather than the de-differentiation of immature/mature vSMCs [15,5]. As both A10 and A7r5 are derived from embryonic tissue, both cell lines were examined for their stem marker expression with a view to investigating whether these vSMC cell lines share characteristics with resident vascular stem cells in culture.

## Materials and methods

### Materials

All materials were of the highest purity commercially available. Primary antibodies included: SMA (monoclonal mouse anti-α-actin antibody, Sigma Cat No: A5228), SM-MHC (monoclonal mouse anti-myosin antibody, Sigma Cat No: clone hSM-V, M7786), (anti-MHC antibody [1G12], Abcam Cat No: Ab683) and (the goat polyclonal MYH11 Antibody (N-16) from Santa Cruz, Cat No: SC79079 ), CNN1 (monoclonal mouse anti-calponin antibody, Sigma Cat No: C2687), Sox10 (monoclonal rabbit anti-Sox10 antibody, Abcam Cat No: ab155279), Sox17 (monoclonal rabbit anti-Sox17 antibody, Millipore Cat No: 09-038) and S100β (monoclonal rabbit anti-S100β antibody, Millipore Cat No: 04-1054), CD44 (polyclonal rabbit anti-CD44, Abcam Cat No: Ab24504), CD29 (monoclonal rabbit anti-CD29, Millipore Cat No: 04-1109), CD146 (monoclonal rabbit anti-CD146, Millipore Cat No: 04-1147), Sca1 (rabbit polyclonal ant-Sca1, Millipore Cat No: AB4336), c-kit (polyclonal rabbit anti-c-Kit, Bioss Cat No: bs-10005R, polyclonal rabbit anti-c-Kit, Santa Cruz Cat No: sc-168) and flt-1 (monoclonal rabbit anti-Flt-1 Abcam Cat No: ab32152) and β-actin (monoclonal mouse anti-β-actin, Sigma Cat No: A5316).

### Cell culture

A10 and A7r5 cells were obtained from ATCC Rockville, MD. Rat aortic SMC [rSMCs, R354-05a] were obtained from Cell Applications, CA. Cells were maintained in either Dulbecco’s Modified Eagle’s Medium (DMEM) or RPMI 1640 media supplemented with 10% foetal bovine serum (FBS), 150 units/ml penicillin, and 150 μg/ml streptomycin (P/S) as previously described [21]. Cells were grown at 37°C in 5% CO_2_ and 95% air. Confluent cells were passaged using 2x trypsin/0.53 mM EDTA. Gibco rat mesenchymal stem cells (MSCs) were obtained from Life Technologies, CA. MSC cells were maintained in growth media made up of 50:50 minimal essential medium (α-MEM) and Ham’s F12 supplemented with 10% MSC defined FBS, 150 unit/ml penicillin, and 150 μg/ml streptomycin. Mesenchymal stem cells (MSCs) were characterised by differentiation along adipogenic lineages and the expression of cell surface markers indicative of MSC (i.e. CD29, CD44, CD90, CD146).

### Isolation of rat multipotent vascular stem cells [MVSCs]

MVSCs were isolated from rat aortic explants as described previously [5]. Briefly, male Sprague Dawley rats were first anesthetized with pentobarbital sodium (0.1 mg/g) and then perfused with 10 mL of PBS. Arterial tissues were harvested as quickly as possible and further dissected in DMEM supplemented with 1% fetal bovine serum (FBS). The aorta was then isolated from the lower thoracic aorta to the upper abdominal aorta. The endothelium was removed by scraping off the cell layer on the luminal surface with sterile scalpel blade before the adventitia was carefully removed from media following brief enzymatic digestion with 2.5 mg/mL of collagenase for 15 min at 37°C using forceps under a dissection microscope. The remaining media was cut into 1-mm pieces, placed onto the surface coated with 1% CellStart (Invitrogen) in 6-well plates and grown in MVSC culture medium containing DMEM with 2% chick embryo extract (MP Biomedical), 1% FBS, 1% N2 (Invitrogen), 2% B27 (Invitrogen), 100 nM retinoic acid (Sigma-Aldrich), 50 nM 2-mercaptoethanol (Sigma-Aldrich), 1% P/S and 20 ngml^-1^ bFGF (R&D Systems) (maintenance medium). All procedures were approved by the University Animal Care Committee and were carried out in accordance with EU guidelines for the Protection of Animals used for Scientific Purposes, (Amendment) Regulations 2013 (S.I. No 434 of 2013).

### Cell proliferation assays

Cells were seeded at 1 × 10^4^ cells/well and quiesced for 48 h. The cells were then grown in DMEM supplemented with 10% FBS over 12 days. At each time point, the cells were washed twice in PBS and fixed in 4% paraformaldehyde (PFA) solution for 10 minutes at room temperature followed by 2 washes of PBS. Following the fixation process, cells were stained for 10 minutes in PBS containing 0.1% Triton X-100 with 30 μM DAPI. Fluorescent images were collected by an Olympus DP-50 fluorescent microscope with the appropriate excitation and emission spectra at 4 ×, 10 ×, 20 × magnification before the fluorescent DAPI labeled nuclei were the counted automatically using the Fiji software package [22].

### Immunoblot analysis

Protein extracts (15-40 μg) were fractionated by SDS-PAGE on 7-15% (w/v) polyacrylamide resolving gels, as previously described [23]. Minor differences in protein loading and transfer were normalised using a Ponceau S stain and by measuring the constitutive β-actin protein levels.

### Quantitative real-time quantitative RT-PCR

Quantitative real-time RT-PCR was performed using the Rotor Gene (RG-3000, Corvett Research) and the SYBR green PCR kit (Qiagen) according to the manufacturer’s instructions. Cells were initially seeded onto 35mm culture dishes at a density of 1 × 10^5^ cells/well in DMEM with 10% FBS, 1% penicillin/streptomycin and quiesced for 24 hours. Cells were then subjected to conditions of serum and serum deprivation in DMEM with 5% FBS, 1% P/S and DMEM with 0.5% FBS, 1% P/S respectively for 48 hours. RNA was isolated using the Maxwell™ 16 Total RNA Purification kit according to the manufacturer’s instructions. RNA samples were quantified and investigated for purity using the nanodrop 2000 spectrophotometer (Thermo Scientific). Total RNA (1-10 ng) was then reverse transcribed and PCR amplified in a one-step reaction containing RT mix, SYBR green mix, and RNase free water at 55°C for 10 mins, 95°C for 5 mins, followed by 60 cycles of 95°C for 5 seconds, 60°C for 15 seconds and subsequent melt curve analysis. Samples were run in triplicate with a no reverse transcriptase (-RT) control. Gene expression was normalised to that of the housekeeping gene, GAPDH.

### Flow cytometry

Cells were cultured until confluency in T75 flasks and then trypsinised in 2x trypsin/0.53 mM EDTA at 37°C. Cells were resuspended in media, counted, and then fixed in BD Cytofix/Cytoperm solution (BD Bioscience) for 20 mins at 4°C. Cells were then washed in 1X BD Perm/Wash solution (BD Bioscience) by centrifugation at 500 g for 3 mins. Following washing, cells were resuspended in 1X BD Perm/Wash solution containing 1 μg of appropriate primary antibody and incubated at 4°C for 30 mins. Following washing, cells were resuspended in 1X BD Perm/Wash solution containing 1 μg of appropriate enzyme-linked secondary antibody and incubated at 4°C for 30 mins. Cells were then washed in BD Perm/Wash solution and resuspended in a final volume of 500 μL of BD Perm/Wash solution.

### Immunocytochemistry

Cells were seeded at a density of 1 × 10^4^ cells/well onto non-coated glass cover slips (20 mm) (Thermo-Scientific) in 35 mm culture dishes and quiesced by serum deprivation for 24 hours. Cover slips were sterilised in IMS and washed twice in PBS prior to culturing. Media was then replaced to media containing either media containing 0.5% or 10% FCS and cells were cultured for a further 48 hours. Cells were washed twice in PBS and fixed for 15 min at room temperature (RT) with 3.7% paraformaldehyde prepared in PBS, washed twice with PBS, and then permeabilised by a 15 min RT exposure to a 0.1% Triton X-100/PBS solution. The cells were washed, blocked for 1 hour with a PBS solution containing 5% BSA and 1% Tween before treatment with specific primary antibodies. Secondary antibodies were Alexa Fluor 546 goat anti-rabbit, rabbit anti-mouse and goat anti-mouse (Invitrogen) and Alexa Fluor 488 goat anti-rabbit (Invitrogen). Nuclei were stained with 4, 6-diamidino-2-phenylindole (DAPI) at a concentration of 2 μg/ml in PBS at RT for 10 minutes. Fluorescent images were collected by an Olympus DP-50 fluorescent microscope with the appropriate excitation and emission spectra at 4 ×, 10 ×, 20 × and 60 × magnification. Non-specific labeling was assessed following secondary antibody treatment.

### Confocal microscopy

A7r5 and A10 cells were plated onto glass coverslips, placed in six well culture plates and returned to the incubator for a minimum of 24 h to allow for cell attachment and spreading. Cells were fixed and permeabilised by the addition of ice-cold acetone for 1 min. The cells were then washed multiple times (3X) with phosphate-buffered saline (PBS) containing 0.5% TWEEN-20 (PBS-T); pH 7.5, and incubated for 10 min in blocking solution (5% non-fat dry milk in PBS-T). Cells were stained for 30 min at room temperature with specific antibodies followed by incubation with an Alexa 488-labelled secondary antibody (Molecular Probes, Eugene, OR, USA). The cells on cover slips were mounted on slides with antifade medium (Dako). Slide preparations were observed using a Zeiss Axio Observer. Z1 equipped with a Zeiss 710 and ConfoCor3 laser scanning confocal head (Carl Zeiss, Inc.). Images were analyzed using Zen 2008 software as previously described [24].

### Adipocyte differentiation

Cells were seeded onto 6-well-plates at a density of 50,000 cells/well. Cells were allowed to recover from trypsinisation for 2 days in complete medium. After recovery, cells were cultured in adipocyte differentiation media for 14d (StemPro® Adipogenesis Differentiation, Life Technologies) as according to the manufacturer’s instructions. Adipocyte differentiation was evaluated by Oil Red O and HCS LipidTOX™ Green neutral lipid staining (InVitrogen), as described by the manufacturers protocols.

### Osteoblast differentiation

Cells were seeded onto 6-well-plates at a density of 50,000 cells/well and allowed to recover from trypsinisation for 2 d in maintenance medium. After recovery, cells were cultured in StemPro® Osteogenesis Differentiation Media Kit, Life Technologies) according to the manufacturer’s instructions. Osteoblast differentiation was evaluated using 2% Alizarin Red S stain (Sigma).

### Notch inhibition

Cells were seeded at a density of 1.5 × 10^4^ cells/well onto non-coated glass cover slips (20 mm) in 35 mm culture dishes and cultured for 72 hours in DMEM containing 10% FBS and 1% P/S. Cells were then treated with either DMSO (0.1%) or the γ - secretase inhibitor, DAPT (10 μM) for 48 hours. Cells were fixed for immunostaining as described above. DAPT, at a stock concentration of 18 mg/ml, was diluted to a 10 μM working solution in DMEM containing 10% FBS and 1% P/S.

### Statistics

Results are expressed as mean ± SEM. Experimental points were performed in triplicate, with a minimum of three independent runs. A t-test was used for comparison of two groups. A value of p ≤ 0.05 was considered significant.

## Results

**Figure 1:**
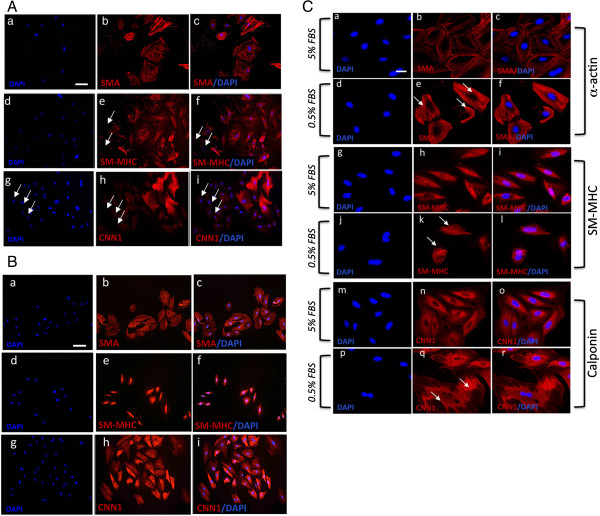
**Vascular SMC Differentiation Marker expression. A** and **B**. Representative immunocytofluorescence of SMC differentiation markers in A10 and A7r5, respectively. Cells were cultured in DMEM supplemented medium for 3 days before immunocytochemistry was performed. Cells stained positive for **(a-c)** SMA **(d-f)** SM-MHC, and **(g-i)** CNN1. The nuclei of were stained with DAPI. Scale bar applies to all images, 250 nM. **C.** Confocal immunocytochemical staining of SMC differentiation markers in A7r5 cells. Quiesced A7r5 cells and grown in DMEM supplemented with **(a-c, g-I, m-o)** 5% FBS and **(d-f, j-l, p-r)** 0.5% FBS for 72 h before cells were analyzed under confocal immunofluorescence microscopy for SMC markers **(a-f)** SMA and **(g-l)** SM-MHC and **(m-r)** CNN1. Arrows designate myofilamentous structures. The nuclei of were stained with DAPI. Scale bar, 100 nm and 250 nm. Data are representative of individual slides with similar results.

A10 and A7r5 cells were cultured in normal DMEM media supplemented with 10% FCS before phenotypic analysis, based on SMC differentiation marker expression, was performed. The embryonic A10 cell line had the typical myoblast morphology when grown in culture and was positive for SMC differentiation markers SMA, CNN1 and SM-MHC (Figure 1A). In addition, every cell appeared positive for each of these antigens. In contrast, embryonic A7r5 cell line had a flat ribbon-like structure and grew to parallel arrays of spindle shaped cells when confluent (Figure 1B and C). Moreover, while these cells were also positive for SMC differentiation markers SMA, CNN1 and SM-MHC, there was a proportion of A7r5 cells that appeared to be weakly positive for SM-MHC and CNN1 when compared to the proportion of SMA positive cells (Figure 1B).

Quiescence of SMCs by serum deprivation has previously been shown to drive SMC differentiation with increased expression of SMA, CNN1 and SM-MHC reported [13]. We evaluated the repercussions of serum-deprivation on A10 and A7r5 SMC differentiation by measuring changes in the distribution and expression of SMC differentiation markers (SMA and CNN1) by confocal microscopy. In the case of A7r5 cells, there was a notable redistribution of SMA and CNN1 filaments in cells following serum-deprivation for 72h when compared to serum conditions (Figure 1C). Confocal immunocytofluorescence demonstrated a marked reorganization of actin filaments under serum conditions, compared with serum deprivation, with less filamentous staining and greater intensity at the periphery (Figure 1C). In a similar manner, the distribution and filamentous nature of CNN1 staining was greater in serum-deprived cells when compared to serum conditions [Figure 1C]. In contrast, there was no significant change in SM-MHC distribution (Figure 1C). A similar profile with regard to the reorganization of SMA and CNN1 filaments and their filamentous nature was evident in A10 cells in response to serum deprivation (data not shown).

**Figure 2:**
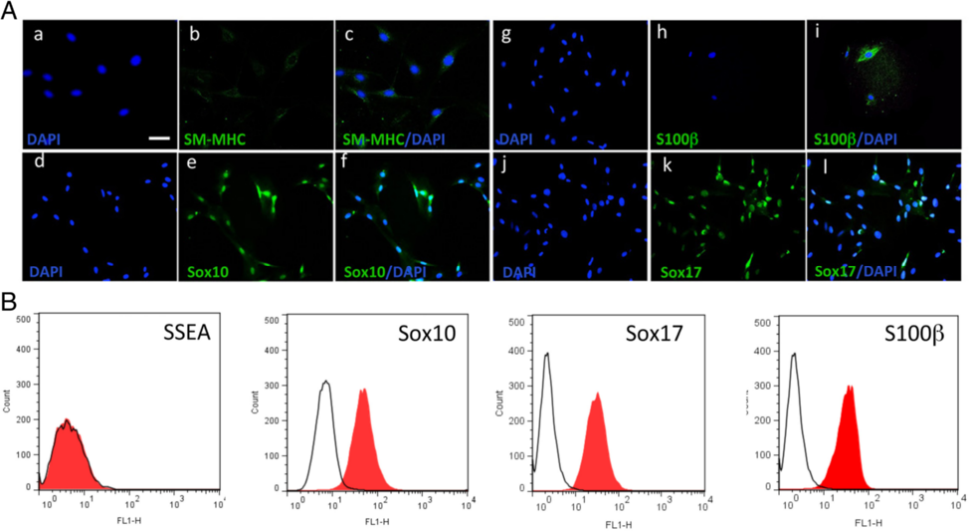
**Multipotent vascular stem cell (MVSC) marker expression. A**. Representative immunocytofluorescence of SMC differentiation markers **(a-c)** SM-MHC and neural stem cell markers **(d-f)** Sox10, **(g-i)** S100β and **(j-l)** Sox17 in markers rat aortic MVSCs isolated by explant and cultured in MVSC maintenance media. The nuclei of were stained with DAPI. Scale bar, 100 nm. Data are representative of three experiments. **B**. Representative flow cytometry analysis of MVSCs cultured in DMEM supplemented with 10% FBS for 10 d with antibodies against SSEA-1, Sox10, Sox17 and S100β. Open curves represent negative control samples; red filled curves represent samples stained with antibodies against SM-MHC, Sox10, Sox17 an S100β.

Previous studies had suggested that SM-MHC negative (SM-MHC^-^) cells can be defined as synthetic and/or proliferative SMCs in culture [4,14]. More recent studies now suggest that SM-MHC^-^ cells derived from the vascular media by explant culture are multipotent vascular stem cells [MVSCs] capable of transition to several different non-vascular lineages [5,15]. Therefore, MVSCs isolated from rat aortic explants as described previously [5], served as our control MVSC population. Microscopic analysis confirmed that the MVSCs were immunocytochemically negative for SM-MHC, but positive for neural stem cell markers Sox10, Sox17 and S100β (Figure 2A). The MVSCs also expressed several MSC-like phenotypic markers CD44 and CD29 in culture but were negative for CD146 [data not shown]. FACS analysis confirmed that the MVSCs were positive for Sox10, Sox17 and S100β (Figure 2B) but negative for the stage specific embryonic antigen (SSEA-1) marker.

**Figure 3:**
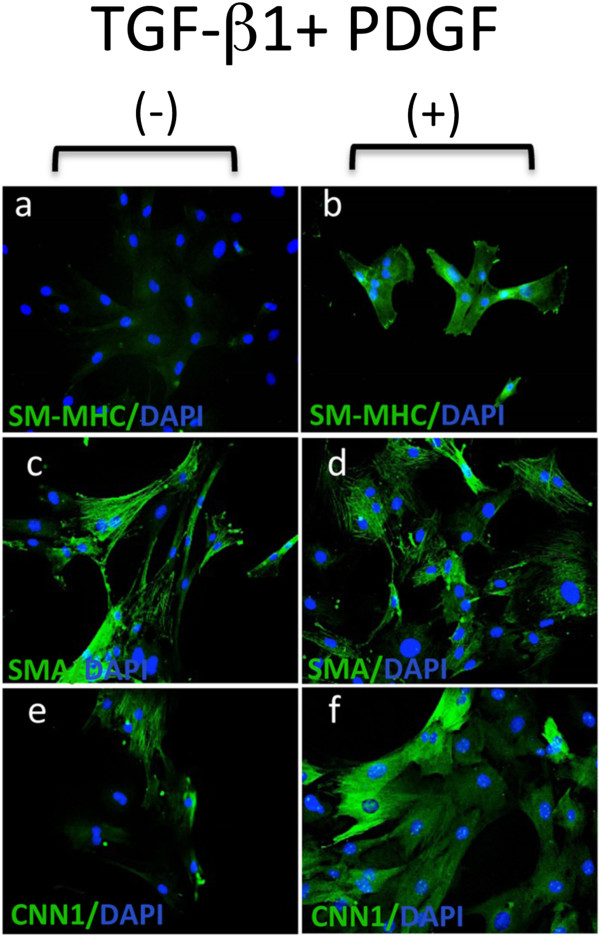
**MVSC transition to vSMC following inductive stimulation.** Representative immunocytofluorescence of SMC differentiation markers **(a, b)** SM-MHC, **(c, d)** SMA and **(e, f)** CNN_1_ in MVSCs cultured in differentiation media (DMEM supplemented with 10% FBS) for 7 d in the absence or presence of 2 ng/ml TGF-β1 and 10 ng/ml PDGF. The nuclei stained with DAPI. Scale bar, 100 nm.

While the MVSCs were immunocytochemically Sox10^+^ and SM-MHC^-^, they expressed SMA in both maintenance and DMEM supplemented media [data not shown]. Moreover, the expression of SMC differentiation markers, SM-MHC and CNN1, was greatly enhanced following culture of these cells for 7 d in DMEM media supplemented with 10% FBS (differentiation media) in the presence of the inductive stimuli, TGF-β1 (2ng/ml) and PDGF (10ng/ml) when compared to differentiation media over the same time period (Figure 3). FACS analysis of MVSCs further confirmed that these cells remain Sox10^+^ and Sox17^+^ positive while concomitantly expressing SM-MHC after 7 d [data not shown].

**Figure 4:**
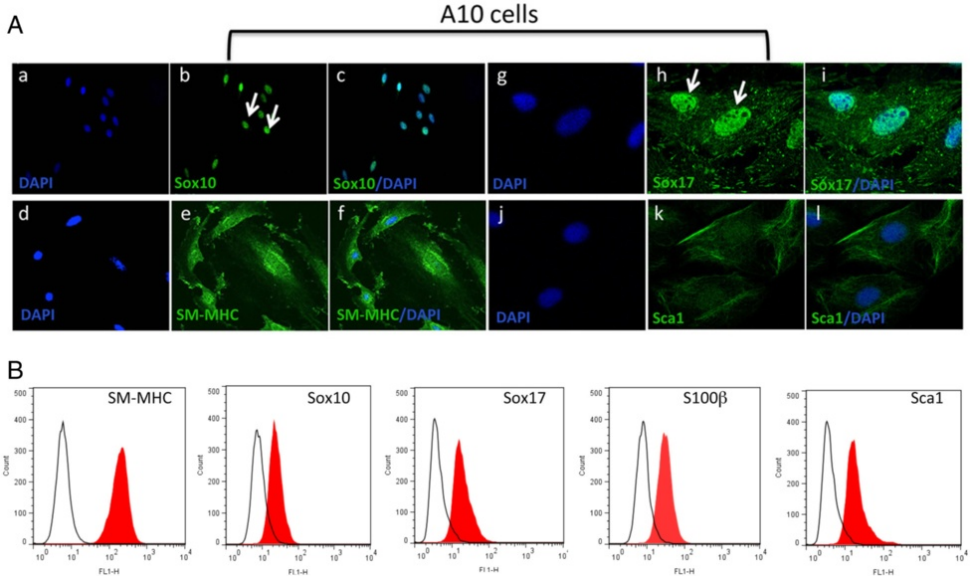
**MVSC marker expression in A10 cells. A**. Representative immunocytofluorescence of neural stem cell markers **(a-c)** Sox10, **(d-f)** SM-MHC **(g-i)** Sox17 and **(j-l)** Sca1 in quiesced cells grown in normal DMEM media supplemented with FBS for 3 d and visualised by **(a-c)** immunocytochemistry **(d-i)** confocal microscopy. Arrows show localisation of Sox10 and Sox17 primarily to the nucleus. The nuclei were stained with DAPI. Scale bar applies to all images, 250 nm except (a-c, 100 nm). Data are representative of three individual slides. **B**. Representative flow cytometry analysis of A10 cells with antibodies against SM-MHC, Sox10, Sox17, S100β and Sca1. Open curves represent negative control samples; red-filled curves represent samples stained with antibodies against SM-MHC, Sox10, Sox17, S100β and Sca1.

We therefore used anti-Sox10 and anti-SM-MHC antibodies to investigate the presence of ‘MVSC-like’ positive cells (SM-MHC^-^ Sox10^+^) in each of the embryonic SMC cell lines. Using immunocytochemistry, we found no evidence for SM-MHC^-^ cells present in A10 cultures (Figure 4). In contrast, all of the cells stained strongly for SM-MHC^+^ and uniformly expressed markers including neural crest cell markers Sox10 and the endoderm marker Sox17 (Figure 4A). These cells were also positive for stem cell antigen, Sca1^+^. Subsequent FACS analysis of SMC differentiation and MVSC marker expression in these A10 cells confirmed quantitatively that >90% of the cells were MHC^+^ Sox10^+^, Sox17^+^, S100β^+^ and Sca1^+^ (Figure 4B).

**Figure 5:**
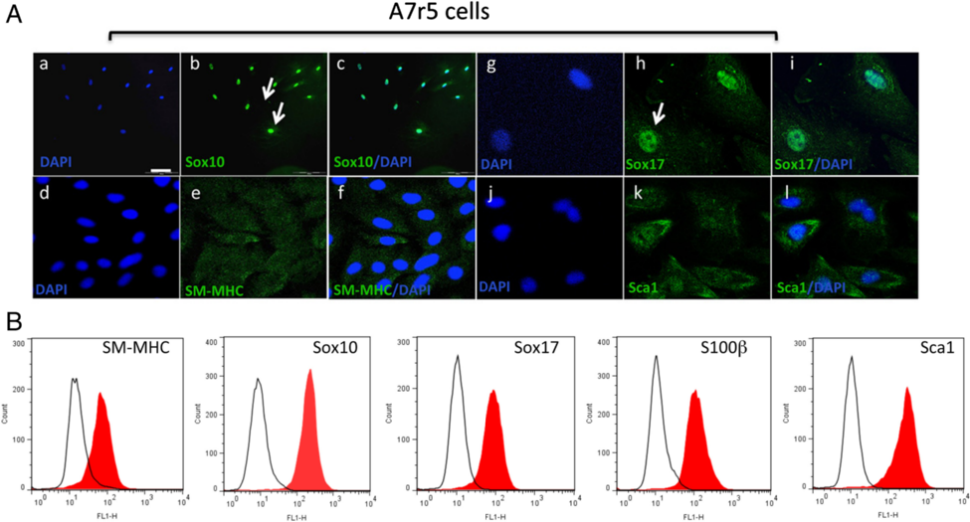
**MVSC marker expression in A7r5 cells. A**. Representative immunocytofluorescence of neural stem cell markers **(a-c)** Sox10, **(d-f)** SM-MHC **(g-i)** Sox17 and **(j-l)** Sca1 in quiesced cells grown in normal DMEM media supplemented with FBS for 3 d and visualised by **(a-c)** immunocytochemistry **(d-i)** confocal microscopy. Arrows show localisation of Sox10 and Sox17 primarily to the nucleus. The nuclei were stained with DAPI. Scale bar applies to all images, 250 nm except (a-c, 100 nm). Data are representative of three individual slides. **B**. Representative flow cytometry analysis of A7r5 cells with antibodies against SM-MHC, Sox10, Sox17, S100β and Sca1. Open curves represent negative control samples; red-filled curves represent samples stained with antibodies against SM-MHC, Sox10, Sox17, S100β and Sca1.

**Figure 6:**
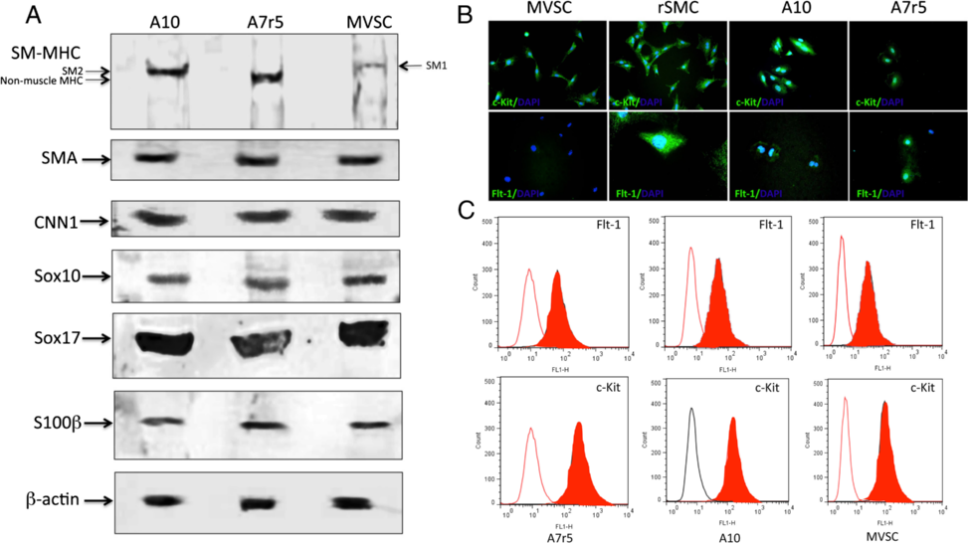
**SMC and stem cell marker expression in A10, A7r5 and MVSCs. A**. Representative immunoblots for SMC differentiation markers (SM-MHC, SMA and CNN1) and neural stem cell markers (Sox10, Sox17, S100β in lysates from quiesced cells grown in normal DMEM media supplemented with FBS for 3 d. The levels of SM-MHC (Sm1, Sm2 and non-muscle MHC), SMA, CNN1, Sox10, Sox17 and S100β were determined using specific antisera against these antigens. Data are representative of blots with similar results. Equal loading was confirmed by Ponceau S staining of the membranes and by measuring the constitutive β-actin gene. **B**. Immunocytochemical staining of c-Kit and Flt-1 in MVSCs, rSMC, A7r5 and A10 cells. Quiesced cells were grown in normal DMEM media supplemented with FBS for 3 d before the cells were stained for c-kit and flt-1. **C**. Representative flow cytometry analysis of Flt-1 and c-Kit in A7r5, A10 and MVSCs using antibodies against Flt-1 and c-Kit. Open curves represent negative control samples; red filled curves represent samples stained with antibodies.

A7r5 cells were also positive SM-MHC, albeit not as robust as A10 cells, and were SM-MHC^+^ by FACS analysis. A7r5 were also positive for neural stem cell markers Sox10^+^, Sox17^+^ and S100β^+^ (Figure 5A). These cells were also positive for Sca1^+^ (Figure 5A). Subsequent FACS analysis confirmed quantitatively that >90% of the cells were MHC^+^ Sox10^+^, Sox17^+^, S100β^+^ and Sca1^+^ (Figure 5B). Parallel Western blot analysis of SMC differentiation and MVSC stem cell marker expression confirmed that both A10 and A7r5 cells express SMA, CNN1 and the MVSCs markers. However, the SM-MHC isoforms for A10 and A7r5 were different. The A10 cells expressed Sm2 whereas the A7r5 cells predominantly expressed non-muscle MHC, as previously reported [16,17]. The MVSCs derived from rat aorta expressed Sm1 [Figure 6A]. In addition, the A10 and A7r5 cells both expressed c-kit and Flt-1 by immunocytochemistry and by FACS analysis (Figure 6B and C). However, unlike A10 and A7r5, MVSC and rSMCs were both positive for c-kit but weakly expressed flt-1 (Figure 6B and C).

**Figure 7:**
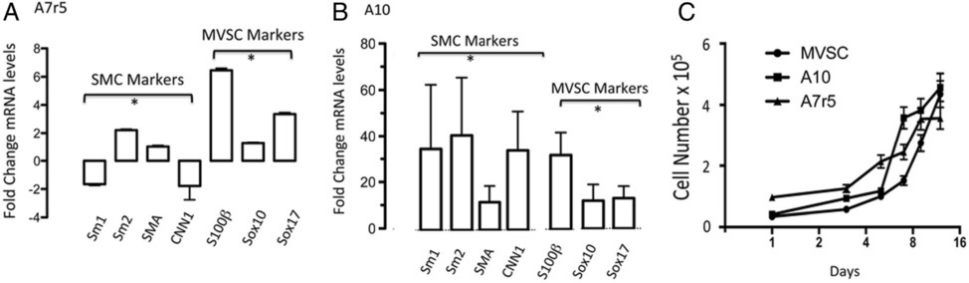
**Quantitative qRT-PCR of neural stem cell marker and SMC differentiation marker mRNA levels. A **and **B**. Quiesced A7r5 and A10 cells were cultured in DMEM media supplemented with 5% FBS and 0.5% FBS for 3 d before relative gene expression of SMC markers [SMA, MHC and CNN1] and neural stem cell MVSC markers [Sox10, Sox17 and S100β] were determined by qRT-PCR. *GAPDH* was used to normalize gene expression. Data are mean ± SEM and are representative of three independent wells, *p < 0.01 when compared to 0.5% FCS. **C**. Representative growth curves for A10, A7r5 and MVSCs grown for 12 d in their respective media. Cell number was quantified by counting DAPI stained nuclei.

To further investigate the expression of MVSC markers within the A10 and A7r5 cell lines, we carried out gene expression analysis of SMC differentiation and MVSC marker expression by real-time qRT-PCR. The repercussions of serum deprivation on SMC differentiation and MVSC marker expression in A7r5 cells were evaluated by measuring changes in SMC differentiation [SMA, Sm1, Sm2 and CNN1] and neural crest stem cell [Sox10, Sox17, S100β] marker gene expression. Serum stimulation resulted in a significant increase in Sox10, Sox17 and S100β for A7r5 cells with a concomitant decrease in the SMC differentiation markers Sm1 and CNN1 while SMA and Sm2 levels increased. The increase in marker expression was greatest for S100β (Figure 7A). In contrast, serum stimulation increased A10 SMC differentiation marker expression with a concomitant increase in neural crest cell markers Sox10, Sox17 and S100β expression in these cells (Figure 7B). In addition, the growth of MVSCs lag behind the A10 and A7r5 in the early growth phase, but recover by day 12 [Figure 7C].

**Figure 8:**
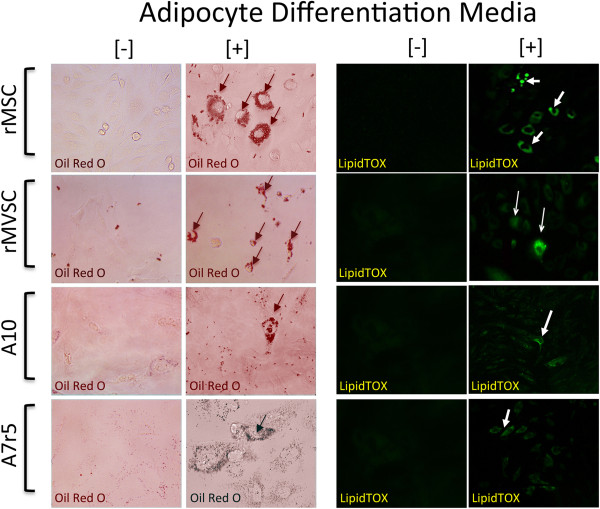
**Multipotent potential of MSC, MVSC, A10 and A7r5 cells *in vitro*.** Representative images of adipocyte differentiation of MSCs, MVSCs and A10 and A7r5 cells following treatment of cells with adipocyte differentiation media for 14 d. Adipocyte differentiation was determined by both Oil Red O and LipidTOX™ staining of lipid droplets. Data are representative of three independent experiments.

Expression of neural stem cell markers [Sox10, Sox17 and S100β] suggests that these embryonic SMC cell lines may retain some stem cell properties. To assess whether A10 and A7r5 cell lines were multipotent, we determined their capability of becoming adipocytes following treatment with specific induction media. Adipocyte differentiation was determined by both oil red staining and lipidTOX™ fluorescent staining after a 14 d treatment with the induction media. Rat mesenchymal stem cells (rMSCs) and rat aortic derived MVSCs (rMVSCs) were also treated with the same adipocyte induction media and served as positive controls (Figure 8). Bovine aortic endothelial cells served as a negative control (data not shown). Both rat MSCs and MVSCs were capable of differentiation to adipocytes after 14d treatment since the number of Oil Red O positive and LipidTOX™ positive cells was significantly increased (Figure 8). In parallel studies, rat MSCs and MVSCs were also capable of differentiation to osteoblasts after 14 d inductive treatment when analyzed with Alizarin Red S [data not shown]. While both A10 and A7r5 cells did retain some Oil Red O stain and LipidTOX™ fluorescent stain following treatment with inductive differentiation media, the number of positive cells was significantly less than MVSCs or the MSCs (Figure 8).

**Figure 9:**
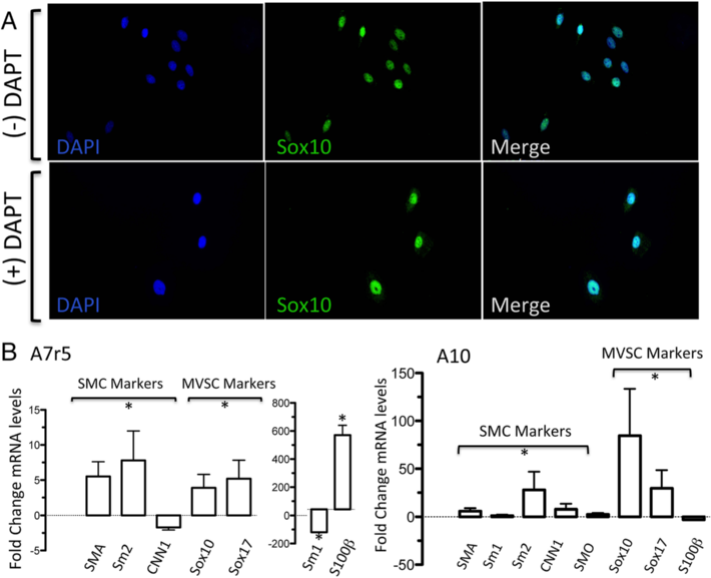
**The effects of Notch inhibition with DAPT on Sox10 expression in A7r5 and A10 cells. A**. Quiesced A10 cells were grown in media containing 10% FBS supplemented with or without 10 μM DAPT for 3 d before the expression of Sox10 was determined by immunocytochemistry. **B**. Relative gene expression of SMC markers [(SMA, MHC, CNN1 and smoothelin (SMO)] and neural stem cell MVSC markers [Sox10, Sox17 and S100β)] in A10 and A7r5 cells was determined by qRT-PCR. *GAPDH* was used to normalize gene expression. Data are mean ± SEM and are representative of three independent wells, *p < 0.01 when compared to 0.5% FCS.

As Notch is a critical arbiter of mesenchymal stem cell (MSC) transition to vascular lineages [25,26] and since MVSC transition to a MSC-like intermediate occurs en route to their differentiation to vSMC [5], we examined the effect of Notch inhibition on A10 and A7r5 differentiation. Notch 1 receptors, like in rat [27] and human vSMC [28], were present on these cells [data not shown]. We investigated if Notch inhibition following treatment with a γ-secretase inhibitor (DAPT), could force these cells to adopt a more contractile phenotype and decrease MVSC marker expression by monitoring SM-MHC and Sox10 expression using immunocytochemistry and qRT-PCR. We found that treatment of both cell types with DAPT did not significantly alter the percentage of Sox10^+^, despite a change in cell number following Notch inhibition (Figure 9A, B). Moreover, Notch inhibition significantly enhanced MVSC marker expression [Sox10, Sox17 and S100β] in both cell types while preferentially increasing Sm2 and SMA in A7r5 but SMA, Sm1, Sm2, SMO and CNN1 in A10 cells (Figure 9A, B).

## Discussion

The characteristics of vSMCs and their modulation in culture have long been the subject of investigation [2,29]. The advent of stem cell derived SMCs has opened up a new debate about the phenotype and origin of ‘modulated’ de-differentiated, neointimal cells *in vivo* and *in vitro*[15,30-32]. Accordingly, we performed a comprehensive analysis of SMC differentiation and MVSC marker profile, growth properties and multipotency capacity for embryonic A7r5 and A10 SMC cells in culture.

The embryonic cell lines A7r5 and A10, and adult aortic SMC cells have all been shown to express SMC differentiation markers including SMA, CNN1, SM22, tropoelastin and to a lesser extent, SM-MHC [17,33]. Interestingly, A10 and to some extent A7r5 resembled the epithelioid phenotype similar to that of the SMC population (“pup cells”) cultured from the intimal thickening 15 days after endothelial injury [34]. A10 and A7r5 were also similar to cloned newborn rat SMC in that they continue to express SMA and SM-MHC in culture [33]. Both cell lines express abundant SMA and CNN1 with similar growth characteristics. Moreover, in agreement with previous studies of cultured SMC, the filamentous profile for SMA and CNN1 was enhanced under conditions of serum deprivation [12]. However, the expression of SM-MHC was noticeably different [17,18]. In the case of A10, the cells predominantly expressed Sm2 with some myofilamentous structures evident under both serum and serum-deprived conditions. In contrast, A7r5 cells predominantly expressed non-muscle SM-MHC with no discernible myofilaments. However, A7r5 have several characteristics in common with neointimal cells including the expression of SMA and non-muscle MHC [16].

The stem cell origin of vSMCs within vascular lesions has been controversial [30-32]. Initial studies in canine carotid arteries suggested that neointimal modulated proliferative SMC were not derived from differentiated cells but instead formed by a myosin negative type 2 medial SMC cell [14]. Since then, several major types of resident SMC-related vascular progenitors have been identified within the tunica media. These include mesenchymal stem cell (MSC)-like cells, a side population of Sca1^+^ progenitors and multipotent vascular stem cells (MVSCs). MSC-like cells express CD29 and CD44 and have multilineage potential for osteogenic and chondrogenic differentiation but importantly, not for adipogenic differentiation [35]. MVSCs on the other hand express neural stem cell markers including Sox17, Sox10 and S100β, are cloneable, have telomerase activity and can differentiate into neural cells and MSC-like cells that subsequently differentiate and transition to SMCs. Sca-1+ progenitors within the adventitia or media can differentiate into SMCs [29] and contribute to atherosclerosis of vein grafts in ApoE-deficient mice [20,36]. Further CD146^+^ perivascular MSC-like cells demonstrate osteogenic, chondrogenic and adipogenic potentials [37].

Importantly, lineage tracing has not resolved whether adult rat SMCs are derived from differentiated medial SMCs [9], MVSCs [5], or both. Elegant epigenetic signature studies tracking histone modifications of the Sm2 (MYH11) locus both *in vitro* and *in vivo* further suggest that SMCs both in culture and following injury are derived from mature differentiated SMC [11,38]. While the passage number, confluency and method of isolation for these SMCs i*n vitro* may impact on these findings, it is clear that epigenetic signature studies are most useful when cells have lost a particular phenotype (and hence specific marker) or changed to multiple phenotypes. However, this is not the case in this study as A10 cells express both MVSC and SMC markers (Sm2 and non-muscle SM-MHC), respectively to varying degrees. Additionally, as MVSCs acquire SM-MHC (Sm2) expression when they are cultured in DMEM 10% FBS media (and not maintenance media [11,38]), or induced using TGF-β1, it is possible that the same histone modifications at the SM-MHC locus are apparent for MVSCs when acquiring SM-MHC expression.

The possibility also exists that de-differentiated SMC derived from differentiated medial SMC may revert back to multipotent/pluripotent stem cells and express neural stem cell markers. Indeed, recent studies have suggested that under certain circumstances, cultured rat SMC can be induced to osteogenic and skeletal muscle lineages [39,40] further supporting a plasticity and stemness associated with SMC in culture that may involve acquiring neural stem cell markers. Indeed somatic cells in general can respond to stimulus-triggered acquisition of pluripotency [STAP] [41,42]. Our study clearly demonstrates that both A10 and A7r5 uniformly express the neural stem cell markers [Sox10, Sox17 and S100β] typical of MVSCs [5] but also express Sca1, which is associated with perivascular adventitial progenitor stems cells [20]. They also concomitantly express SMC differentiation markers, SMA and CNN1 and Sm2 and non-muscle MHC, respectively [42]. Our data also suggest that both A10 and A7r5 cells also maintain some multipotent capabilities as they mimic MVSCs and MSC’s in their ability to differentiate to adipocytes (and osteoblasts), albeit less robustly, following inductive stimulation. In addition, DAPT which inhibits Notch signalling and is a critical component during MSC to SMC transition [26,43,44] significantly alters both MVSC and SMC differentiation markers in both cell lines promoting neural stem cell phenotypes while increasing SMC differentiation. We have previously shown that Notch promotes SMC de-differentiation *in vitro*[28]. It is also worth noting that mechanistically, Sox17 acts upstream of the Notch system and downstream of the canonical Wnt system in orchestrating arterial specification during development and may thus be critical for MVSC transition to SMC *in vitro*[45].

The reason(s) for the appearance of MVSC neural stem cell markers in SMC cell lines in culture remain(s) unknown. One possibility is that both cell lines originate from MVSCs where the SMCs from MVSCs outgrow the de-differentiated SMC and eventually dominate the cultures [15]. In this regard, the growth curves for MVSCs, A10 and A7r5 suggest that MVSCs lag behind the embryonic cells in the early growth phase, but recover to comparable growth rates by day 12. Notwithstanding the controversy as to whether neointimal SMC following injury are derived from resident stem cells or de-differentiated SMCs, or even both, there is no controversy surrounding the phenotype of medial SMC in normal vessels *in situ* before culture. In this context, SMC prepared by explant culture from SM-MHC-Cre/LoxP-enhanced green fluorescence protein (EGFP) mice are predominantly immunocytochemically eGFP negative (and hence SM-MHC negative) but Sox10, Sox17 and S100β positive, yet acquire eGFP (SM-MHC) when sub-cultured *in vitro* or activated by a Notch ligand or TGF-β1 *in vitro*[5]*.* In contrast, medial vSMC before enzymatic dispersal are predominantly eGFP (SM-MHC) positive and express little to no Sox10, Sox17 or S100β in situ. This profile is maintained when cells are enzymatically dispersed and cultured at P0 [15]. Over time, these cells are widely thought to become ‘modulated’ and reduce their expression of SMC differentiation markers, in particular SM-MHC, when compared to cells in situ or P0 in culture. Our data for rat aortic derived MVSCs suggests that cells maintained in stem cell maintenance media at early passage are predominantly SM-MHC^-^ (Sm2) negative but positive for Sm1, Sox10^+^, Sox17^+^ and S100β^+^. Importantly, they can transition to adipocytes (and osteoblasts) following specific inductive stimulation and enhance their expression of SMC differentiation markers when grown in non-MVSC stem cell media (DMEM + 10% FCS or TGF-β1).

The presence of stem cell antigen, Sca1^+^ on both embryonic SMC lines suggest that these cells also exhibit some perivascular markers. Vascular Sca1^+^ is associated with adventitial progenitors and a side population of medial progenitors that have the capacity of differentiate to SMC [6,20,29,36]. While MVSCs are reported to be initially Sca1^-^ negative [5], the relationship, if any between MVSCs in the media and Sca1^+^ progenitors in the adventitia remains unknown. In addition, the presence of c-kit and Flt-1 positive cells within the A10, A7r5 and MVSC population is characteristic of SMCs derived from large arteries of older vessels and may suggest a similar stem cell origin [46].

In conclusion, the expression of neural stem cell markers in A10 and A7r5 suggests that these SMCs may represent MVSC-derived SMC, de-differentiated SMC or both. In addition, their expression of perivascular Sca1 suggests that these cells may also have adventitial mesoderm origins. Both cell lines should now prove useful in determining the functionality of SMC in disease as they clearly resemble neo-intimal SMC known to be derived from MVSC following vascular injury [5]. Further single cell tracing experiments will be required to delineate the precise relationship between these two origins.

## Competing interests

On behalf of all authors, the corresponding author states that there is no competing interests.

## Authors’ contributions

EK, RH, CG and EM carried out the cell isolation, characterization and marker expression studies on each cell line. EK, CG, CM carried out the qRT-PCR analysis. EK, EM and SG performed the FACS analysis. EM, LEC and CEL carried out the confocal microscopic analysis. SG, DM EMR and PAC participated in the supervision of experiments and data analysis. EMR and PAC participated in the design, drafting and writing of manuscript. All authors have read and approved the final manuscript.
